# Ad-VT oncolytic adenovirus suppresses bladder cancer via cAMP-dependent AMPK-Raptor activation and G2/M arrest

**DOI:** 10.1016/j.tvr.2026.200337

**Published:** 2026-01-29

**Authors:** Dapeng Li, Jing Lu, Ran Zhu, Xianyan Sun, Cuiling Zhang, Mingzhe Sun, Chengyuan Ma, Chao Shang, Xiao Li

**Affiliations:** a Department of Neurosurgery, The First Hospital of Jilin University, Changchun, 130012, China; b Changchun Veterinary Research Institute, Chinese Academy of Agricultural Sciences, Changchun, 130122, China; c Animal Disease Prevention and Control Center of Helong City, Yanbian Korean Autonomous Prefecture, 130000, China; d Medical College, Yan Bian University, Yanji, 130000, China

**Keywords:** Bladder cancer, Apoptin, Oncolytic adenovirus, Cell cycle arrest, AMPK signaling pathway

## Abstract

Bladder cancer remains a leading cause of cancer-related mortality with limited therapeutic options. This study investigates the antitumor efficacy and mechanism of Ad-VT, a dual-specific oncolytic adenovirus expressing apoptin under the hTERT promoter, in bladder cancer. *In vitro*, Ad-VT selectively killed bladder cancer cells (UM-UC-3, T24, 5637, RT4) while sparing normal urothelial cells (SV-HUC-1), showing dose-dependent cytotoxicity (70 % inhibition at 100 MOI in 5637 cells). It induced G2/M phase arrest via downregulation of cyclin B1/cdc2 and upregulation of p-cdc2/p21. Mechanistically, Ad-VT elevated cAMP levels, activating the AMPK-Raptor-mTOR pathway. This was confirmed by pathway inhibitors (Dorsomorphin, ESI-09) and siRNA knockdown, which reversed cell cycle arrest and reduced cytotoxicity. *In vivo*, intratumoral Ad-VT injection suppressed UM-UC-3 xenograft growth, enhanced survival, and increased apoptosis while reducing proliferation. Crucially, AMPK inhibition attenuated Ad-VT's antitumor effects. These results demonstrate that Ad-VT exerts potent, tumor-selective activity against bladder cancer by inducing cAMP-dependent AMPK-Raptor-mTOR signaling and G2/M arrest, supporting its therapeutic potential.

## 1 Introduction

Bladder cancer remains one of the leading causes of cancer-related mortality, as current clinical treatments demonstrate limited therapeutic efficacy [1]. The persistent risk of postoperative recurrence and disease progression continues to compromise patient quality of life, highlighting an urgent need for novel therapeutic strategies [2]. Oncolytic virotherapy demonstrates clinical potential by utilizing naturally occurring or genetically engineered viruses that selectively replicate in cancer cells, express therapeutic genes to eliminate malignancies while preserving normal tissue. This approach directly lyses tumor cells through viral proliferation. In 2015, the U.S. FDA approved T-VEC (talimogene laherparepvec), a genetically modified herpes simplex virus type 1, as the first oncolytic virus therapy for metastatic melanoma, marking a landmark achievement in cancer therapy [3].

In previous studies, we developed a dual-specificity oncolytic adenovirus, Ad-Apoptin-hTERTp-E1A (Ad-VT), engineered with three tumor-targeting components: the adenoviral backbone, apoptosis-inducing apoptin protein, and tumor-specific regulatory elements (hTERT promoter and E1A gene). Ad-VT selectively infects cancer cells, undergoes robust replication, and expresses apoptin to induce tumor cell death. Preliminary investigations demonstrated its potent antitumor activity across multiple malignancie, with notable efficacy against bladder cancer [[4], [5], [6]]. However, the precise molecular mechanisms underlying Ad-VT's anti-bladder cancer effects remain incompletely elucidated.

Apoptin, the protein product of the VP3 gene of chicken anemia virus, possesses a wide array of anti-tumor properties, specifically causing apoptosis in transformed or tumor cells while leaving normal cells unaffected [7]. Typically localized in the nucleus of tumor cells and predominantly found in the cytoplasm of normal cells, phosphorylated Apoptin can translocate to the nucleus, leading to protein interactions that instigate tumor cell apoptosis [8]. Nevertheless, due to Apoptin's inability to penetrate tumor cells independently, a suitable carrier becomes necessary [9]. The carrier vector must not only effectively transport and express Apoptin in malignant cells but also must ensure non-toxicity towards normal cells. To address this, an adenovirus expression vector was developed that contained the hTERTp-E1a expression cassette, leveraging the functionalities of hTERT and Apoptin to generate a bispecific oncolytic adenovirus (Ad-VT) tailored for tumor cells. Ad-VT capitalizes on tumor-specific replication and targeted cell death, presenting a promising avenue for tumor gene therapy [10].

Our previous study demonstrated that AD-VT induces bladder cancer cell death by triggering autophagy [11]. Additionally, research indicates that the alphavirus-based oncolytic virus M1 enhances cytotoxicity against refractory cancer cells through cyclic adenosine monophosphate (cAMP) pathway activation, which elevates cyclin-dependent kinase 2 (CDK2) phosphorylation levels, arresting cells in the S phase and promoting apoptosis [12,13]. Building on these findings, we investigated AD-VT's impact on the cell cycle and confirmed its ability to upregulate cAMP expression, activate the AMPK-mTOR signaling axis, and modulate cell cycle progression, thereby exerting anti-bladder cancer effects. These results position AD-VT as a promising therapeutic intervention for bladder cancer. While the impact of Ad-VT on bladder cancer cell cycles remains unexplored, this study delves into investigating its effects on cell cycle dynamics and signaling pathways in this context.

This study investigated the anti-tumor effects and mechanisms of Ad-VT on bladder cancer both *in vitro* and *in vivo*. The findings indicate that Ad-VT can arrest bladder cancer cells at the G2/M phase of the cell cycle and modulate the cell cycle by upregulating the cAMP-activated AMPK-mTOR signaling pathway, leading to anti-tumoral effects. Moreover, the research offers foundational experimental data endorsing Ad-VT as a promising therapeutic intervention for bladder cancer.

## 2 Results

### 2.1 Efficiency of Ad-VT infection of cells and Ad-VT inhibits bladder cancer cell proliferation in *vitro* and suppresses tumor growth in *vivo*

**Fig. 1:**
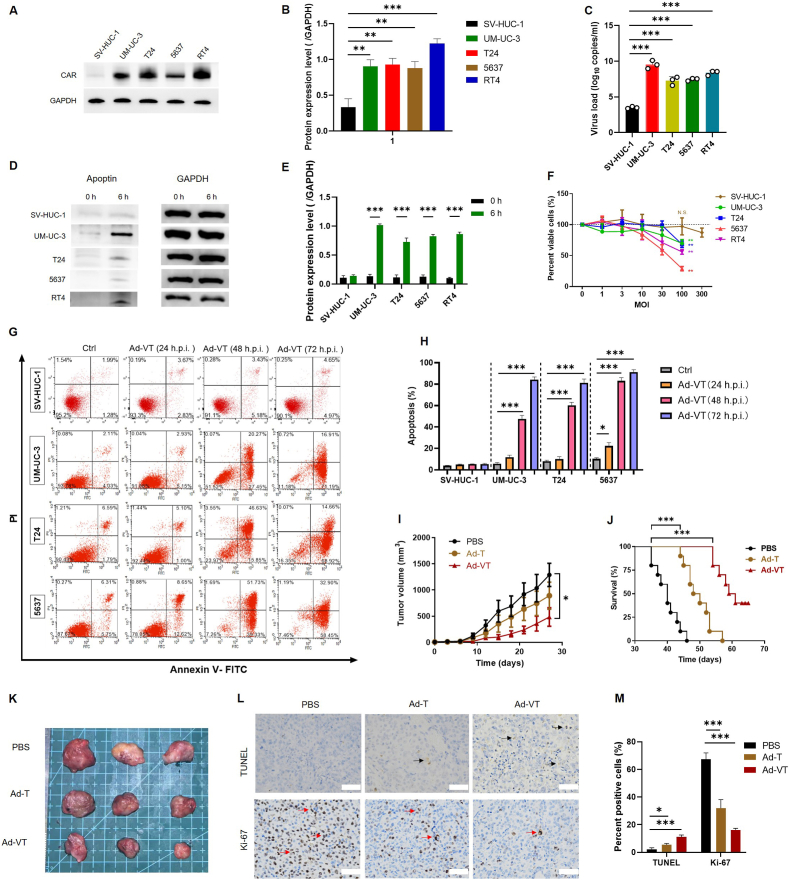
Efficiency of Ad-VT infection in cells and antitumoral effect of Ad-VT on bladder cancer cells in *vitro* and *in vivo*. (A, B) Western blot analysis of CAR receptor expression levels in SV-HUC-1, UM-UC-3, T24, 5637, and RT4 cell lines. (C) qPCR detection of adenovirus viral load in SV-HUC-1, UM-UC-3, T24, 5637, and RT4 cell lines after 6 h of Ad-VT infection. (D, E) Western blot analysis of Apoptin protein expression levels in SV-HUC-1, UM-UC-3, T24, 5637, and RT4 cell lines after 6 h of Ad-VT infection (MOI = 100). (F) Cell viability was assessed using the Cell Counting Kit-8. The specified cells were infected with Ad-VT at various concentrations (MOI = 0, 1, 3, 10, 30, 100, 300) for 48 h (G, H) Flow cytometry analysis was carried out to determine the percentage of apoptotic cells at 0, 24, 48, and 72 h post Ad-VT infection (MOI = 100). (I, J, K) Tumor growth curves, survival curves, and representative tumor images of mice were illustrated. (L, M) TUNEL assay and Ki-67 staining in PBS or Ad-VT treated tumors were presented. The data are shown as mean ± SEM, with statistical significance denoted as ∗*p* < 0.05, ∗∗*p* < 0.01, ∗∗∗*p* < 0.001.

Previous studies have indicated that the level of CAR expression in bladder cancer cells directly influences the transduction efficiency of adenoviruses [14]. Therefore, we detected CAR protein expression levels in SV-HUC-1, UM-UC-3, T24, 5637, and RT4 cells by Western blot, respectively. As shown in Fig. 1A and B, it was found that the CAR protein expression levels in bladder cancer cells were higher compared to the normal epithelial cell line SV-HUC-1. The high expression of CAR receptors on bladder cancer cells provides a prerequisite for the infection and entry of the oncolytic adenovirus Ad-VT. Subsequently, we evaluated the uptake levels of Ad-VT in normal urothelial cells and bladder cancer cells. After infecting SV-HUC-1, UM-UC-3, T24, 5637, and RT4 cells with Ad-VT for 6 h, the viral load was detected by qPCR to reflect the cellular uptake level of Ad-VT. The results (Fig. 1C) showed that the uptake level of the oncolytic adenovirus in bladder cancer cells was significantly higher than that in normal epithelial cells, with UM-UC-3 cells exhibiting the highest uptake of Ad-VT. Meanwhile, the expression level of the apoptin protein was detected by Western blot (Fig. 1D and E). The results indicated that the expression levels of apoptin were significantly elevated in UM-UC-3, T24, 5637, and RT4 cells, with the highest expression observed in UM-UC-3 cells. This suggests that Ad-VT has strong replication and protein expression capabilities in these cells.

To evaluate the selective inhibitory effects of Ad-VT on bladder cancer cells, we treated bladder cancer cell lines (UM-UC-3, T24, 5637, and RT4) and an immortalized bladder epithelial cell line (SV-HUC-1) with graded concentrations of Ad-VT for 48 h, followed by assessment of cell viability using the CCK-8 assay. Experimental results revealed that 48-h Ad-VT treatment significantly reduced the viability of four bladder cancer cell lines in a dose-dependent manner. Notably, no inhibitory effects were observed in SV-HUC-1 normal urothelial cells. At a concentration of 100 MOI, Ad-VT achieved nearly 70 % inhibition in 5637 cancer cells, whereas even at 300 MOI, it exhibited no significant cytotoxicity toward SV-HUC-1 cells, demonstrating tumor-selective therapeutic efficacy. These results suggest that Ad-VT exerts a targeted cytotoxic effect on bladder cancer cells in a dose-dependent manner, while maintaining non-toxicity towards normal bladder cells (Fig. 1F).

Ad-VT is a genetically engineered oncolytic adenovirus incorporating an apoptin expression cassette. Apoptin, the apoptosis-inducing protein derived from chicken anemia virus (CAV), selectively triggers programmed cell death in malignant cells while sparing normal tissues [15]. To further investigate the impact of Ad-VT on apoptosis in bladder cancer cells, flow cytometry was utilized. It was observed that treatment with 100 MOI of Ad-VT led to significantly elevated levels of apoptosis in SV-HUC-1, UM-UC-3, T24, and 5637 cells at 48 h and 72 h compared to their corresponding control groups. The apoptotic rates in these cell lines progressively increased with time (Fig. 1B), reaching approximately 9.62 % for SV-HUC-1 cells, 88.1 % for UM-UC-3 cells, 83.58 % for T24 cells, and 91.35 % for 5637 cells at 72 h in the Ad-VT treated group. Ad-VT treatment did not lead to a significant increase in the apoptosis rate of SV-HUC-1 cells. These values were markedly higher than those in the control groups (Fig. 1G and H), underscoring the ability of Ad-VT to induce robust apoptosis in bladder cancer cell.

To assess the *in vivo* effect of Ad-VT on bladder cancer, a subcutaneous tumor-bearing model of UM-UC-3 cells was created in nude mice. Compared to Ad5, Ad-T contains the hTERT-E1A sequence, and Ad-VT includes apoptin in addition to Ad-T. Therefore, comparing Ad-T and Ad-VT helps evaluate the antitumor effect of Apoptin. Tumor size was measured every 3 days after successful tumor establishment. The results showed that the tumor-killing effect of the Ad-T group was stronger than that of the PBS group but significantly weaker than that of the Ad-VT group. Ad-VT demonstrated the best antitumor effect. As shown in Fig. 1I and J, at the endpoint of treatment, the average tumor volume in the Ad-VT treatment group was reduced by approximately 65 % compared to the PBS group (*p* < 0.05), and the survival of the mice was significantly prolonged. Subsequently, tumor tissues were subjected to TUNEL staining and Ki-67 immunohistochemical staining. Representative microscopic images (Fig. 1L) and quantitative analysis results (Fig. 1M) showed that, compared to the PBS control group, the Ad-VT and Ad-T treatment groups exhibited enhanced tumor cell apoptosis and reduced proliferative activity. These findings indicate that Ad-VT has the ability to impede the growth rate of UM-UC-3 tumors *in vivo*. These outcomes suggest that Ad-VT treatment enhances the survival rate of nude mice and exhibits an antitumoral effect *in vivo*. Consistent with its *in vitro* pro-apoptotic and anti-proliferative effects on bladder cancer cells. These results strongly support the therapeutic potential of Ad-VT for bladder cancer treatment.

### 2.2 Ad-VT induces cell cycle arrest of UM-UC-3 cells at G2/M phase

**Fig. 2:**
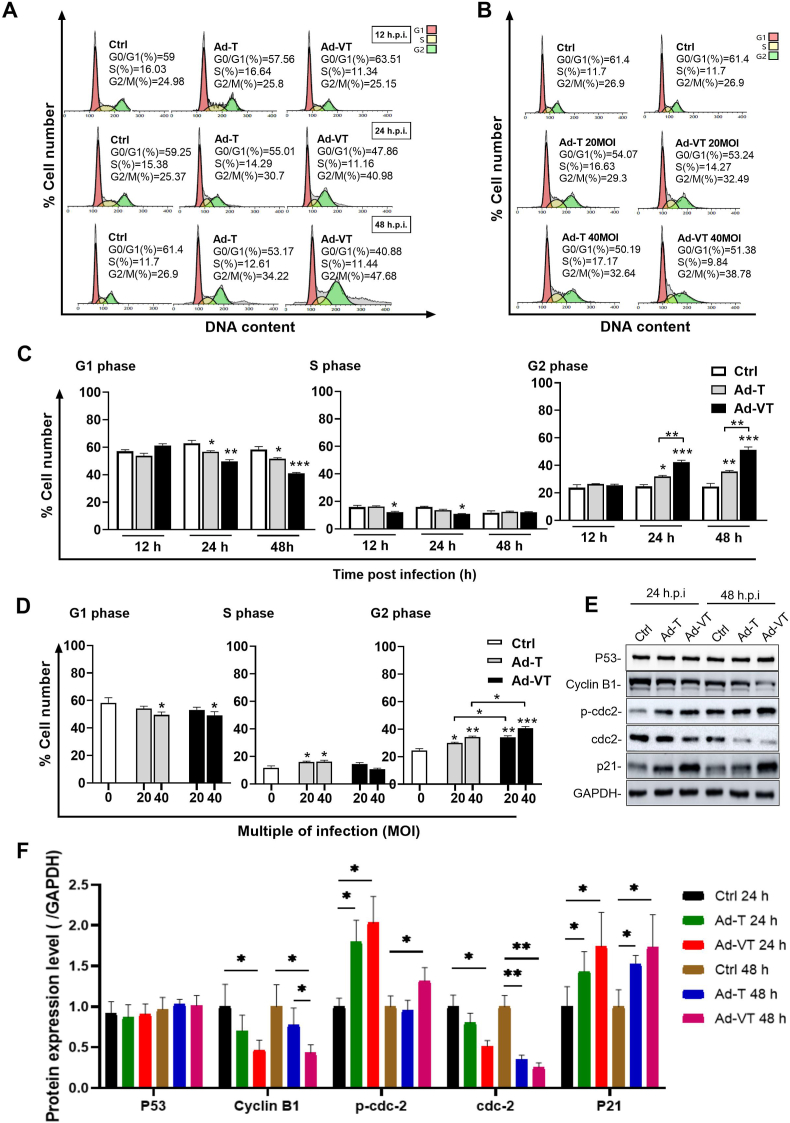
Ad-VT induces cell cycle arrest of UM-UC-3 cells at G2/M phase. Ad-T:Ad-hTERTp-E1A Ad-VT: Ad-Apoptin-hTERTp-E1A **(A, C)** The distribution of the cell cycle and analysis of UM-UC-3 cells infected with Ad-VT was conducted at various time points (MOI = 100). **(B, D)** The cell cycle distribution of UM-UC-3 cells infected with Ad-VT was analyzed at various MOIs, 24 h post-infection. **(E, F)**The expression levels of cell cycle-related proteins in UM-UC-3 cells 24 h post-infection with Ad-VT (MOI = 100). The data are presented as mean ± standard error of the mean (SEM), and statistical significance is denoted as ∗*p* < 0.05, ∗∗*p* < 0.01, and ∗∗∗*p* < 0.001.

Research has demonstrated that viruses frequently manipulate cell cycle progression to establish cellular conditions most favorable for their replication [16]. To investigate the role and mechanism of apoptin in inhibiting the proliferation of UM-UC-3 cells, we examined the cell cycle distribution of SV-HUC-1 cells and UM-UC-3 cells following infections with Ad-T and Ad-VT. The key distinction between Ad-T and Ad-VT lies in the presence of the apoptin gene cassette, which is exclusively and Ad-VT at 20, 40 and 100 MOI, respectively, and cell cycle analysis was performed at 12h, 24h, and 48 h post-infection. Flow cytometry analysis demonstrated that both Ad-T and Ad-VT treatment induced significant G2/M phase arrest in UM-UC-3 cells in a time- (Fig. 2A, C and Supplementary Fig. 1A and B) and dose-dependent manner (Fig. 2B, D and Supplementary Fig. 1C and D) compared to control. Additionally, we performed corresponding experiments in the control cell line SV-HUC-1. After infection with Ad-VT at MOIs of 20, 40, and 100 for 24 h, cell cycle analysis was conducted. The results, presented in Supplementary Fig. 1C and D, demonstrated that Ad-VT did not induce cell cycle arrest in SV-HUC-1 cells. Adenovirus vector alone (Ad-T) can indeed induce a certain degree of G2/M phase arrest. Moreover, we evaluated the impact of Ad-Mock and Ad-apoptin on cell cycle arrest, as shown in Supplementary Fig. 2A and B. The results indicate that Ad-apoptin exhibits a significantly ability to induce G2/M phase arrest in cells compared to Ad-Mock at an MOI of 100.

To validate these observations, we performed Western blot analysis to quantify key G2/M phase regulatory proteins including cyclin B1, p-cdc2, cdc2 and p21. The data revealed significant downregulation of G2M-phase driver proteins (cyclin B1 and cdc2) concomitant with upregulation of inhibitory markers (p-cdc2 and p21) (Fig. 2E and F), consistent with the observed G2/M arrest [13]. Ad-T and Ad-VT effectively induced G2/M phase arrest in UM-UC-3 cells by downregulating cdc2 and cyclin B1 expression while upregulating p-cdc2 and p21 expression. In addition, while p21 was significantly upregulated, the expression level of p53 protein did not exhibit significant changes, indicating that adenovirus infection did not markedly affect the intracellular p53 protein expression level. Additionally, we detected p53 protein expression levels in SV-HUC-1, UM-UC-3, T24, 5637, and RT4 cells. As shown in Supplementary Fig. 3A and B, SV-HUC-1 cells exhibited the highest p53 expression, while UM-UC-3, T24, 5637, and RT4 cells showed significantly lower p53 levels, with T24 and RT4 displaying the lowest expression. This finding is consistent with previous studies demonstrating that apoptin can induce tumor cell apoptosis through a p53-independent pathway [17,18]. Both Ad-T and Ad-VT induced G2/M phase arrest in UM-UC-3 cells through coordinated downregulation of cyclin B1/cdc2 and upregulation of p-cdc2/p21. This cell cycle blockade led to mitotic arrest and subsequent tumor cell death. Notably, Ad-VT exhibited significantly stronger G2/M arrest than Ad-T, suggesting apoptin-mediated synergistic enhancement of the oncolytic effect.

These findings suggest that Ad-VT triggers G2/M phase arrest in UM-UC-3 cells through the combined effects of Apoptin and adenovirus vectors.

### 2.3 Ad-VT induces UM-UC-3 cell cycle arrest by upregulating cAMP

**Fig. 3:**
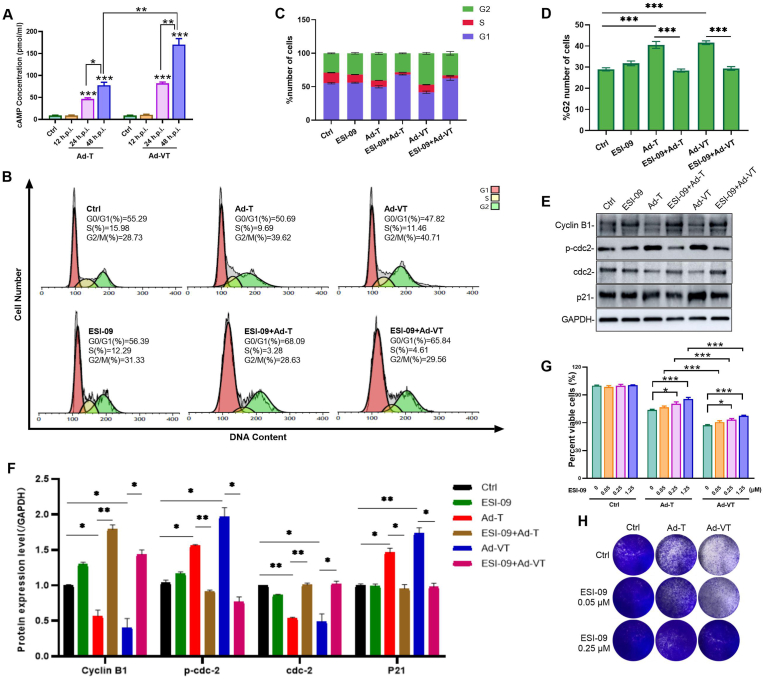
Ad-VT affects UM-UC-3 cell cycle arrest by upregulating cAMP. **(A)** The cAMP content changes following Ad-T and Ad-VT infection of UM-UC-3 cells were measured using ELISA (MOI = 100). **(B, C)** Cell cycle distribution of UM-UC-3 cells treated by ESI-09 (1.25 μM) before viral infection. After 48h of infection, the cell cycle was analyzed by flow cytometry (MOI = 100). **(D)** The proportion of UM-UC-3 cells at the G2 phase in (B). **(E, F)** Western blot analysis of cell cycle-related protein changes in UM-UC-3 cells treated with ES-09, following infection for 48 h (MOI = 100) (**G, H)** UM-UC-3 cells were treated with ESI-09 and then infected with virus for 48 h. Viability assay (G) and crystal violet staining (H) (MOI = 100). Data are presented as mean ± SEM, ∗*p* < 0.05, ∗∗*p* < 0.01, ∗∗∗*p* < 0.001.

Previous studies have indicated that cAMP inhibits cell division and promotes cell differentiation in normal cells by regulating the cell cycle [19]. It is known that an elevated intracellular cAMP concentration impedes cell proliferation. In this study, a cAMP ELISA kit was utilized to measure cAMP levels in UM-UC-3 cells following infection with Ad-T and Ad-VT. The results demonstrate a significant increase in cAMP levels in both Ad-T and Ad-VT treated groups compared to the control group (blank cell group). This increase was observed to be gradual and was positively associated with the duration of infection (Fig. 3A). These findings suggest that Ad-VT can induce a substantial elevation in cAMP content in UM-UC-3 cells, with higher levels seen in the Ad-VT group compared to the Ad-T group. The analysis confirms that the tumor-inhibitory impact of Ad-VT stems from the adenovirus and Apoptin gene, leading to stronger effects than those observed with the Ad-T adenovirus vector.

The increase in cAMP content likely altered the cell cycle of UM-UC-3 cells. Subsequently, we employed the cAMP inhibitor ESI-09 to assess the distribution of the cell cycle post-viral treatment. We observed a significant reversal in the distribution of UM-UC-3 cells at the G2/M phase in the Ad-VT and Ad-T infection groups upon treatment with ESI-09, leading to a significant reduction in cells in the G2/M phase (Fig. 1, Fig. 3B–D). To corroborate this observation, we examined the expression levels of G2/M phase regulatory proteins via Western blot analysis. The results indicated that treatment of cells in the Ad-VT and Ad-T groups with ESI-09 led to a reversal in the expression levels of cdc-2, Cyclin B1, p-cdc2, and p21 proteins (Fig. 3E and F). These findings suggest that the induction of G2/M phase arrest by Ad-VT is linked to cellular cAMP levels, and that inhibition of cAMP can reverse the G2/M phase arrest triggered by Ad-VT.

To investigate the impact of cAMP on the antitumoral efficacy of Ad-VT, experiments utilizing CCK-8 and crystal violet staining were conducted. The findings indicate that both Ad-VT and Ad-T notably diminish cell viability in UM-UC-3 cells. Notably, the cytotoxic effects of Ad-VT and Ad-T on UM-UC-3 cells were markedly attenuated following treatment with the cAMP inhibitor, ESI-09 (Fig. 3G and H). These results suggest that the activation of cAMP can enhance the antitumor effect of Ad-VT in bladder cancer.

### 2.4 Ad-VT induced cAMP upregulation activates the AMPK signaling pathway

**Fig. 4:**
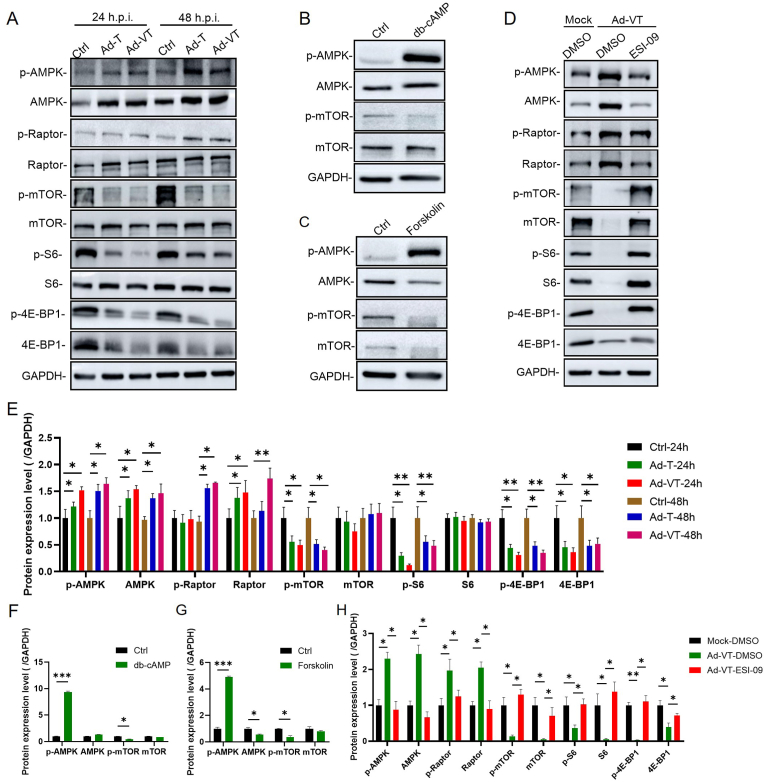
**Ad-VT activates the AMPK signaling pathway by upregulating cAMP. (A, E)** The AMPK-mTOR pathway was analyzed by Western blot. Ad-T and Ad-VT infected UM-UC-3 cells were harvested at different time points (24h and 48 h) (MOI = 100). **(B, F)** Regulation of AMPK and mTOR by db-cAMP. **(C, G)** Regulation of AMPK and mTOR by Forskolin. **(D, H)** Changes in the AMPK-mTOR pathway of Ad-VT infected UM-UC-3 cells that were pretreated with ESI-09 (MOI = 100). Data are presented as mean ± SEM, ∗*p* < 0.05, ∗∗*p* < 0.01, ∗∗∗*p* < 0.001.

The cAMP-AMPK signaling pathway can modulate AMPK activity, a second messenger responsible for intercellular communication and various biological effects [20]. AMPK, an AMP-activating protein, functions to negatively regulate the cell cycle, thereby impeding tumor proliferation [21]. Western analysis indicated that Ad-T and Ad-VT induce the AMPK-mTOR signaling pathway and enhance AMPK expression in UM-UC-3 cells (Fig. 4A–E). To further explore the link between cAMP and the AMPK signaling pathway, two cAMP activators, db-cAMP (a cAMP analog commonly used *in vitro*) and Forskolin (a universal eukaryotic adenylate cyclase activator elevating intracellular cAMP levels), as well as the cAMP inhibitor ESI-09 (an acyclic nucleotide inhibitor that suppresses cAMP activity) were employed. Treatment with db-cAMP and Forskolin significantly increased AMPK expression (Fig. 4B–F) and activated the AMPK-mTOR signaling pathway (Fig. 4C–G), while ESI-09 reduced AMPK expression and inhibited the AMPK-mTOR signaling pathway (Fig. 4D–H). These findings suggest that the inhibitory effect of Ad-VT on the G2/M phase of UM-UC-3 cells resulted from the activation of the AMPK signaling pathway mediated by cAMP upregulation.

### 2.5 Ad-VT-mediated AMPK pathway activation leads to cell cycle progression blockade

**Fig. 5:**
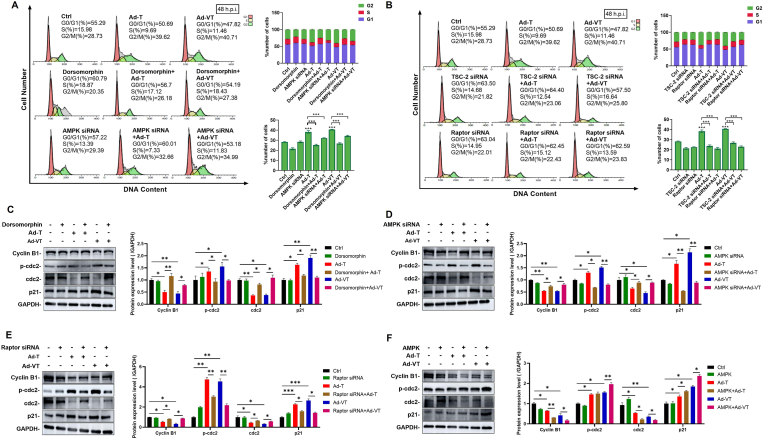
Ad-VT activates AMPK to block cell cycle progression. **(A, B)**The cell cycle distribution of UM-UC-3 cells were detected by flow cytometry. UM-UC-3 cells were first treated with Dorsomorphina (1.25 μM) or transfected with siRNA, and then infected with the viruses for 48 h (MOI = 100). **(C**–**E)** Western blot detection of the expression levels of cell cycle related proteins showing the inhibition of the AMPK pathway, following infection for 48 h (MOI = 100). **(F)** Western blot detection of the expression levels of cell cycle related proteins showing the activation of the AMPK pathway, following infection for 48 h (MOI = 100). Data are presented as mean ± SEM, ∗*p* < 0.05, ∗∗*p* < 0.01, ∗∗∗*p* < 0.001.

AMPK serves as a central metabolic guardian that orchestrates diverse cellular processes—including glycolysis, cell cycle progression, and mitochondrial dynamics—to maintain energy homeostasis [22]. To confirm the involvement of the AMPK pathway in Ad-VT-induced G2/M phase arrest, we utilized the AMPK pathway inhibitor, Dorsomorphin, and assessed its impact on the cell cycle using flow cytometry. Our findings demonstrate that treating Ad-VT and Ad-T infected cells with Dorsomorphin, AMPK siRNA, TSC2 siRNA, and Raptor siRNA led to a significant decrease in the proportion of cells in the G2/M phase (Fig. 5A and B), indicating that Ad-VT induces G2/M phase arrest via the AMPK pathway. Subsequent analysis of cyclin effects revealed that AMPK upregulation increased the expression of G2/M phase regulatory proteins, while Dorsomorphin, AMPK siRNA, and Raptor siRNA reversed this effect (Fig. 5C–F). These results suggest that Ad-VT may impact the cell cycle by modulating the AMPK-mTOR signaling pathway.

### 2.6 Inhibitory effect of Ad-VT on UM-UC-3 cells *in vitro* and *in vivo* through the AMPK-Raptor pathway

**Fig. 6:**
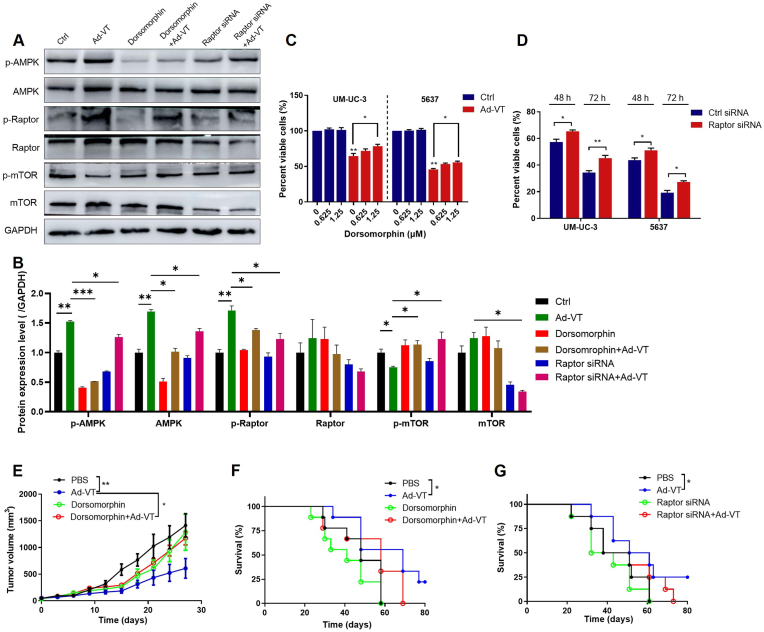
Inhibitory effect of Ad-VT on UM-UC-3 cells *in vitro* and *in vivo* through the AMPK-Raptor pathway. **(A, B)** The expression levels of p-AMPK, AMPK, p-Raptor, Raptor, p-mTOR, and mTOR at 24h after Ad-VT infection (MOI = 100) of UM-UC-3 cells and pretreatment (2h) with a vehicle, Dorsomorphin (1.25 μM) or transfection with the control or Raptor siRNA. **(C, D)** The viability of UM-UC-3 cells and 5637 cells that were pretreated with Dorsomorphin (B) or Raptor siRNA (C). **(E, F)** Effect of Dorsomorphin on tumor volume (D) and survival rate (E) of nude mice treated with PBS or Ad-VT. **(G)** The effect of Raptor siRNA on survival rate of nude mice treated with PBS or Ad-VT treatment. Data are presented as mean ± SEM, ∗*p* < 0.05, ∗∗*p* < 0.01, ∗∗∗*p* < 0.001.

The study further confirmed the involvement of the AMPK-Raptor pathway in Ad-VT-induced cell cycle arrest. Western blot analysis revealed that the AMPK inhibitor Dorsomorphin or Raptor siRNA significantly reversed the expression of AMPK pathway proteins induced by Ad-VT infection (Fig. 6A and B). Additionally, CCK-8 assay was employed to assess changes in the cell viability of UM-UC-3 cells following pretreatment with Dorsomorphin and Raptor siRNA, and subsequent infection with Ad-VT for 48 h or 72 h. Results indicated that Dorsomorphin and Raptor siRNA partially diminished the oncolytic activity of Ad-VT (Fig. 6C and D), indicating that Ad-VT manifests an anti-bladder cancer effect *in vitro* through the AMPK-Raptor pathway.

To investigate AMPK-dependent tumor suppression *in vivo*, we treated T24 xenograft-bearing nude mice with Dorsomorphin (AMPK inhibitor) and Raptor siRNA. As illustrated in Fig. 6E and F, Ad-VT markedly decreased tumor volume and enhanced the survival rate of nude mice. Treatment with 10 mg/kg Dorsomorphin partially attenuated this effect, suggesting a correlation between the *in vivo* anti-tumor activity of Ad-VT and the AMPK pathway. Likewise, Raptor siRNA produced similar outcomes in Ad-VT-treated nude mice (Fig. 6G), indicating that the anti-bladder cancer efficacy of Ad-VT hinges on the AMPK signaling pathway in an *in vivo* setting.

## 3 Discussion

**Fig. 7:**
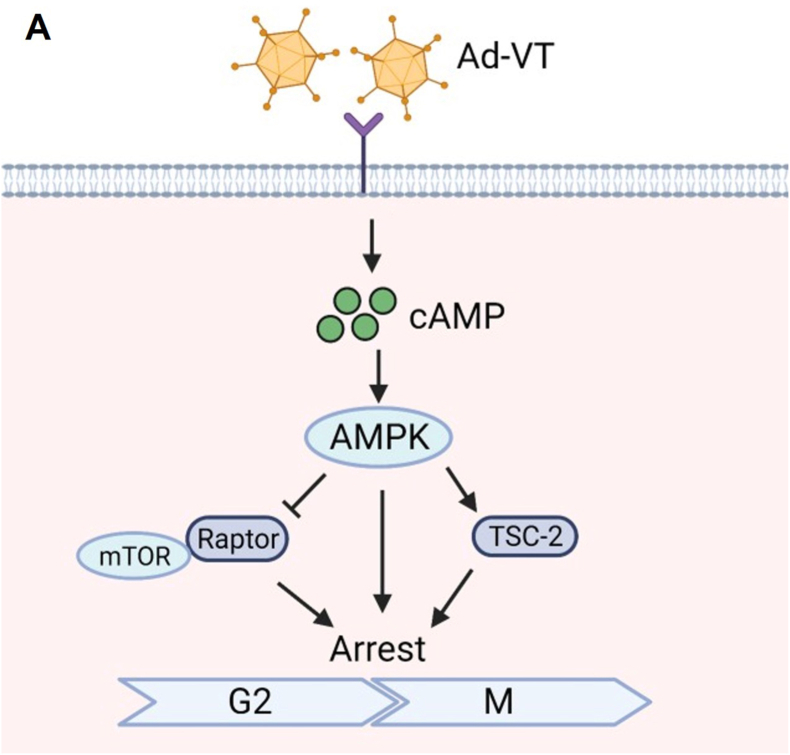
Mechanism of Ad-VT induced activation of G2/M cycle arrest **(A)** The oncolytic adenovirus Ad-VT, which carries apoptin, not only replicates specifically in UM-UC-3 bladder cancer cells, but also induces potent cell death. This effect is primarily mediated by a G2/M phase arrest, which is triggered through the upregulation of cAMP and subsequent activation of the AMPK signaling pathway.

Apoptin, derived from the CAV, is an apoptosis-inducing protein known for its ability to selectively eliminate tumor cells while preserving normal cells [23,24]. Human telomerase reverse transcriptase (hTERT) is pivotal in telomerase regulation, a process absent in most normal human cells [25]. Conversely, hTERT expression and telomerase activation are detected in up to 90 % of human malignancies, contributing to the immortality of tumor cells [26]. Our laboratory developed the oncolytic adenovirus Ad-VT, comprising the hTERT promoter and the tumor-specific apoptin gene. Our designed Ad-VT combines dual mechanisms of “oncolysis” and “apoptin”. The oncolytic virus lyses cells, while apoptin induces profound apoptosis within tumor cells, and the two can generate a synergistic effect. This construct has demonstrated the capacity to trigger apoptosis exclusively in tumor cells without harming healthy counterparts, exhibiting promising inhibitory effects on lung and prostate cancers in preliminary investigations [27,28]. This study evaluated the effect of Ad-VT on bladder cancer cell proliferation and its underlying mechanism. Ad-VT elevates cAMP levels to activate the AMPK signaling pathway and induces G2/M phase arrest, with both effects synergistically driven by the adenovirus and apoptin, thereby enhancing its antitumor efficacy (Fig. 7).

Previous studies have demonstrated Ad-VT's ability to selectively eliminate MCF-7 breast cancer cells, causing abnormal cell cycle alterations characterized by a notable increase in the G1 phase cell population after 24 h of infection [6]. The study investigated the anti-bladder cancer effect of Ad-VT, showing selective effects on the replication and apoptosis of bladder cancer cell lines UM-UC-3, T24, 5637, and RT4 cells, while no effects were observed on normal bladder cell lines (SV-HUC-1). To account for the limitations of *in vitro* assays without a tumor microenvironment, a subcutaneous tumor-bearing model in nude mice was employed, yielding results consistent with the *in vitro* findings [29]. Although 5637 cells showed higher sensitivity to Ad-VT *in vitro*, their low and variable tumor formation in mice led us to select UM-UC-3 cells for more stable *in vivo* studies. In 5637 cells, Ad-VT dose-dependently enhanced apoptosis and induced cell cycle arrest, as shown by flow cytometry. These results are provided in Supplementary Fig. 4A–D. Following intratumoral injection of Ad-VT, a significant reduction in tumor volume and an improvement in survival rate were noted in UM-UC-3 subcutaneous tumor-bearing mice. Tunel and Ki-67 staining confirmed that Ad-VT injection increased apoptotic cells and reduced cell proliferation in tumor tissue. Therefore, Ad-VT demonstrates a substantial anti-bladder cancer effect both *in vitro* and *in vivo*, exhibiting high specificity and safety profile, making it a promising oncolytic virus for bladder cancer treatment.

Previous research has demonstrated that Apoptin in transformed cells associates with subunit 1 of the APC/C complex, a late cell cycle promoting complex. This association leads to cell cycle arrest at the G2/M phase and the initiation of apoptosis independent of p53 [30]. Similar to Apoptin, the adenovirus E4orf 4 protein also induces G2/M phase cell cycle arrest and apoptosis in the absence of p53 [31]. To understand the process of oncolytic adenovirus Ad-VT-induced cycle arrest in bladder cancer, we evaluated the induction of G2/M arrest by Ad-VT on bladder cancer cells in a time- and dose-dependent manner using flow cytometry [32]. Adenovirus vector alone induced some G2/M arrest, and Ad-apoptin showed more pronounced arrest than Ad-Mock. Thus, the G2/M arrest by Ad-VT likely results from synergy between the adenovirus and apoptin. Subsequently, we examined the impact of Ad-VT on the expression of cell cycle regulatory proteins through Western blot analysis. Our results indicated that Ad-VT downregulates Cyclin B1 and cdc2 expression, suggesting that Ad-VT inhibits the levels of these proteins in a time-dependent manner, Ad-T and Ad-VT effectively induced G2/M phase arrest in UM-UC-3 cells by downregulating cdc2 and cyclin B1 expression while upregulating p-cdc2 and p21 expression. Although studies have shown that the first-generation oncolytic adenoviruses were designed based on the principle of selective replication in p53-deficient cells, we also detected P53 protein expression levels in SV-HUC-1, UM-UC-3, T24, 5637, and RT4 cells [33]. As shown in Supplementary Fig. 3A and B, SV-HUC-1 cells exhibited the highest P53 expression level, while the P53 protein expression levels in UM-UC-3, T24, 5637, and RT4 cells were significantly lower than in SV-HUC-1, with T24 and RT4 showing the lowest levels. However, the Ad-VT virus we constructed not only carries the oncolytic adenovirus vector itself but also contains the apoptin gene. Infection of UM-UC-3 cells with Ad-VT resulted in a significant upregulation of p21 without markedly altering p53 protein expression levels, consistent with previous findings that apoptin induces tumor cell apoptosis through a p53-independent pathway [17,18]. Adenovirus infection typically leads to the degradation of p53. However, exceptions exist in certain cases. For instance, infection with E1B-55K gene-deficient adenovirus, as documented in the literature, does not result in p53 degradation [34,35]. Additionally, adenovirus infection does not lead to the degradation of p53 when infecting cells with mutated p53 [[36], [37], [38]]. In this study, the UM-UC-3 cell line used is a p53-mutant type, where the p53 protein is inactive and loses its function, thereby unable to initiate effective antiviral apoptosis. Therefore, the p53 level remains unaffected by adenovirus infection [39,40]. The above results indicate that Ad-VT thereby disrupts the cell cycle progression of UM-UC-3 bladder cancer cells by inducing G2/M phase cell cycle arrest.

The second messenger cAMP is formed following the activation of adenylate cyclase, which catalyzes the conversion of ATP associated with the activity of specific hormones or other signals [41]. It plays a role in regulating the cell cycle and is implicated in tumor formation. The level of cAMP is regulated by the balance between adenylate cyclase and phosphodiesterase. It has been reported that the E1A protein in adenovirus can directly bind to cAMP, inducing the activation of its downstream pathway [42,43]. In this study, we quantified cAMP levels in bladder cancer cells using ELISA and observed a significant increase in cAMP content upon treatment with Ad-VT. Given that cAMP can impede cell proliferation and differentiation, the findings suggest that Ad-VT exerts an inhibitory effect on the proliferation of bladder cancer cells. Furthermore, our results indicated that the cAMP levels in the Ad-VT group exceeded those in the Ad-T group, suggesting that both the adenovirus and Apoptin have antiproliferative effects on bladder cancer cells. To investigate the impact of the cAMP inhibitor ESI-09 on the cells, we conducted assays including flow cytometry, Western blotting, CCK-8, and crystal violet staining. Our results revealed that G2/M phase arrest was reversed and the cytotoxicity of bladder cancer cells induced by the viruses was mitigated. This implies that the G2/M phase arrest induced by Ad-VT is cAMP-dependent, and the cAMP inhibitor has an antagonistic effect on the cell cycle, leading to a significant reduction in the cycle arrest of bladder cancer cells and diminishing the antitumor efficacy of Ad-VT.

Since Ad-VT can induce G2/M phase arrest in bladder cancer cells, we further explored the underlying mechanism. Our investigation revealed that UM-UC-3 bladder cancer cells infected with Ad-VT activate the AMPK signaling pathway leading to an increase in AMPK expression. Given that the cAMP-AMPK pathway plays a crucial role in regulating AMPK activity and subsequent biological outcomes, we subjected Ad-VT to treatment with cAMP activators (db-cAMP and Forskolin) and its inhibitor, ESI-09. Our results demonstrated that cAMP activators significantly stimulate the AMPK signaling pathway, whereas the cAMP inhibitor effectively suppresses this pathway. Therefore, our findings suggest that the inhibitory impact of Ad-VT on bladder cancer cells results from the upregulation of cAMP and subsequent activation of the AMPK signaling pathway.

Modulation of the AMPK signaling pathway has been shown to induce cell cycle arrest [44]. In our study, we utilized various techniques, including the use of an AMPK inhibitor, siRNA, AMPK overexpression, Raptor siRNA, and TSC2 siRNA. AMPK is a critical cellular energy sensor, which is activated in response to a decline in energy status [45,46]. The stress-induced upregulation of AMPK expression enhances its signaling output through increased protein accumulation, leading to broad pathway activation that synergistically contributes to the antitumor effect. Our findings demonstrate that inhibiting the AMPK signaling pathway significantly diminishes the cell cycle arrest triggered by Ad-VT-induced G2/M phase. Conversely, our results also indicate that AMPK overexpression enhances G2/M phase arrest.

To investigate the involvement of the AMPK signaling pathway in Ad-VT-induced antitumor activity, we treated Ad-VT infected bladder cancer cells with Dorsomorphin and Raptor siRNA. Our findings revealed that these treatments partially inhibited the oncolytic activity of Ad-VT. Subsequently, a nude mouse subcutaneous tumor-bearing model was utilized for validation. The outcomes demonstrated that Ad-VT treatment significantly reduced tumor volume and enhanced the survival rate of mice. Conversely, treatment with Dorsomorphin or Raptor siRNA hindered this effect. Furthermore, we delineated that the G2/M phase arrest triggered by Ad-VT relies on the AMPK-Raptor signaling pathway to exert its anti-tumor efficacy.

Studies have indicated that long-term oncolytic virus therapy may lead to drug resistance [[47], [48], [49]]. We isolated and cultured single cells from xenograft tumors, then infected them with Ad-VT and performed cell viability assays. The results, as shown in Supplementary Fig. 5A, demonstrated that compared to the immortalized cell line UM-UC-3, Ad-VT still inhibited the growth of xenograft tumor cells UM-UC-3-X, indicating that UM-UC-3-X did not develop resistance to Ad-VT. However, since this study did not include long-term passaging experiments for validation, we hypothesize that resistance may potentially emerge after prolonged oncolytic virus treatment. Additionally, beyond bladder cancer, Ad-VT may also exhibit therapeutic effects against other tumors, such as lung cancer and ovarian cancer [50,51]. Potential mechanisms may involve disruption of mitochondrial pathways, among others. These findings suggest that Ad-VT holds promising application prospects.

In summary, our study demonstrates that Ad-VT exhibits a notable antitumor impact on bladder cancer by reducing cdc2 and CyclinB1 levels, leading to G2/M phase arrest in UM-UC-3 cells. Additionally, Ad-VT induces anti-bladder cancer effects through the upregulation of cAMP expression and activation of AMPK signaling to regulate the cell cycle. These findings establish a solid groundwork for considering Ad-VT as a viable treatment option for bladder cancer.

## 4 Methods and materials

### 4.1 Viruses, cell lines and animals

The adenoviruse Ad-Mock (E1a-depleted) and the recombinant adenoviruses Ad-Apoptin (E1a-depleted with a CMV-Apoptin insert), Ad-T (Ad-hTERT-E1a) and Ad-VT (Ad-hTERT-E1a-Apoptin) were constructed and stored in our laboratory at the Changchun Veterinary Research Institute, Chinese Academy of Agricultural Sciences in Changchun, China [10]. SV-HUC-1 cells, HEK-293 cells, as well as human bladder cancer cells (UM-UC-3, T24, 5637, and RT4) were obtained from the Cell Bank of the Shanghai Institute for Biological Sciences in Shanghai, China. SV-HUC-1 cells were cultured in Ham's F-12 nutrient medium, UM-UC-3, and HEK-293 cells were cultured in DMEM medium, while T24, 5637, and RT4 cells were cultured in RPMI-1640 medium. All media were supplemented with 10 % fetal bovine serum (FBS), 50 U/mL penicillin, and 50 U/mL streptomycin, and cells were maintained in an incubator at 37 °C with 5 % CO2.

Female BALB/c nude mice (6 weeks old, weighing 22 ± 2 g) were procured from the Beijing SPF Laboratory Animal Technology Company for generating the xenograft tumor model. All experimental procedures were conducted in adherence to the NIH Guide for the Care and Use of Laboratory Animals.

### 4.2 Chemicals and antibodies

Nocodazole (M1404), ESI-09 (SML0814), and dbcAMP (D0627) were purchased from Sigma-Aldrich. Dorsomorphin (HY-13418 A), TC11 (HY-129478), and Forskolin (HY-15371) were purchased from MedChemExpress. All chemicals were dissolved in DMSO and stored at −80 °C. The dilution of the corresponding working concentrations was performed using FBS-free DMEM medium. The primary antibodies, CyclinB1 (#4131), p-cdc2 (#4539), cdc2 (#9116), p21 (#2947), AMPK (#5831), p-AMPK (#2535), Raptor (#2280), p-Raptor (#2083), mTOR (#2983), p-mTOR (#5536), S6 (#2317), p-S6 (#4858), 4E-BP1 (#9644), p-4E-BP-1 (#2855), and GAPDH (#5175) were purchased from Cell Signaling Technology. CAR(Cat No. 86186-1-RR) and P53(Cat No.10442-1-AP)were purchased from Proteintech Group Inc. Apoptin (ab193612)were purchased from Abcam Plc. The secondary antibodies (A0208) were purchased from Beyotime Biotechnology.

### 4.3 qPCR analysis

Real-time quantitative PCR was performed using the TaqMan probe method. The adenovirus-specific primer sequences were: AQ1 (5′-GCCACGGTGGGGTTTCTAAACTT-3′) and AQ2 (5′-GCCCCAGTGGTCTTACATGCACATC-3′). The probe sequence was [6-FAM]TGCACCAGACCCGGGCTCAGGTACTCCGA [TAMRA] [52,53].

### 4.4 CCK-8 assay

Cell viability was determined by the CCK-8 assay, according to the manufacturer's tructions. The cells were counted and seeded at 5 × 10^3^ cells/well in 96-well plates. After 24 h of incubation, the viral or drug treatment was performed. After further incubation for the indicated time (24h, 48h, or 72h), 10 μL of the CCK-8 reagent (Dojindo Molecular Technologies, CK04) was added to each well and incubated at 37 °C for 4 h. The OD at 450 nm was measured using a multifunctional enzyme marker. All drug treatments were added 2 h before viral treatment. In the siRNA treatment group, the cells were first seeded in 6-well plates and transfected with siRNA. After 48 h, these cells were re-seeded into 96-well plates and treated as described previously.

### 4.5 Annexin V-fitc/PI analysis

Cell viability was assessed using the CCK-8 assay following the manufacturer's rotocol. A total of 5 × 10^3^ cells were seeded per well in 96-well plates. Following a 24-h incubation period, treatment with either virus or drug was initiated. After incubation for the specified durations (24h, 48h, or 72h), 10 μL of CCK-8 reagent (Dojindo Molecular Technologies, CK04) was added to each well and incubated at 37 °C for 4 h. The optical density at 450 nm was measured using a multifunctional enzyme microplate reader. Drug treatments preceded viral treatments by 2 h. For the siRNA treatment group, cells were initially seeded in 6-well plates and transfected with siRNA. After 48 h, these cells were transferred to 96-well plates and subjected to the aforementioned treatments.

### 4.6 Cell cycle analysis

UM-UC-3 cells were plated in 6-well plates at a density of 5 × 10^5^ cells per well and incubated for 24 h. Following the treatment of each experimental group, the cells were harvested, fixed with 1 mL of 70 % ice-cold ethanol at 4 °C for 2 h, and subsequently washed with PBS buffer. The cells were then resuspended in 500 μL of dilution buffer, to which 25 μL of propidium iodide (PI) and 2.5 μL of RNase were added. The mixture was incubated at 37 °C for 30 min in darkness, agitated, and filtered through a 300-mesh nylon sieve before analysis by flow cytometry using Kaluza software (Dojindo Molecular Technologies, C543) in Fullerton, CA, USA.

### 4.7 Crystal violet staining

UM-UC-3 cells were seeded in 12-well plates and incubated for 24 h. Various drug treatments were administered 2 h before and 48 h after infection with Ad-T or Ad-VT at a multiplicity of infection (MOI) of 100. Subsequently, 500 μL of 0.4 % crystalline violet staining solution was added to each well and incubated for 10 min. Following incubation, the staining solution was removed, the wells were washed thrice with phosphate-buffered saline (PBS), and then allowed to air dry before being photographed.

### 4.8 Plasmid transfection

Raptor siRNA (sc-44067) and AMPK siRNA (siG000005562 A) were designed and synthesized by Santa Cruz Biotechnology, Inc. The AMPK (FH2213) plasmid was designed and synthesized by Fenghui Biotechnology, Inc. And validated by sequencing. UM-UC-3 cells were seeded in 6-well plates for 24 h. Raptor siRNA was transfected into the cells using Lipofectamine® RNAiMAX (Invitrogen, 13778) and the AMPK plasmid was transfected using Lipofectamine™ 3000 (Invitrogen, L3000008).

### 4.9 G0/G1 and G2/M UM-UC-3 cell synchronization

UM-UC-3 cells were seeded in 6-well plates and incubated for 24 h. Subsequently, the medium was replaced with serum-free culture for a 16-h period to synchronize the cells in the G0/G1 phase. Following this, the cells were treated with 60 ng/ml of Nocodazole for 16 h to synchronize them in the G2/M phase for downstream analysis of viral titer and cell cycle progression.

### 4.10 Virus titers assay

Virus titers were determined using TCID50. Cell culture supernatants from cell cycle-synchronized cells infected with Ad-VT or Ad-T were collected and subjected to 10-fold serial dilutions before infecting HEK-293T cells. Eight replicate wells were established for each dilution, and the cells were monitored daily. The TCID50 values were calculated using the Reed-Muench method.

### 4.11 cAMP level analysis by ELISA

UM-UC-3 cells were seeded in 6-well plates and incubated for 24 h. Subsequently, the cells were infected with Ad-VT at a multiplicity of infection (MOI) of 100. The cell culture supernatants were harvested at 12, 24, and 48 h post-infection. The alterations in cyclic adenosine monophosphate (cAMP) levels pre- and post-viral infection were assessed following the manufacturer's rotocol of the cAMP assay kit (R&D Systems, KGE002B).

### 4.12 Western blot analysis

The cells were harvested, and total cellular proteins were extracted using the Minute™ Total Protein Extraction Kit (Invent Biotechnologies, SD001/SN002). Subsequently, 30 μg of total protein was subjected to SDS-PAGE and transferred onto PVDF membranes. The membranes were then incubated with 5 % skim milk at room temperature for 2 h, followed by an overnight incubation with the primary antibody at 4 °C, and a 40-min incubation with an HRP-labeled secondary antibody at room temperature.

### 4.13 Xenograft model and treatment

Female SPF nude mice (BALB/c, 6 weeks old, weighing 22 ± 2 g) were housed in a sterile environment to establish a bladder cancer transplantation tumor model. UM-UC-3 cells (3 × 10^6/100 μL) were subcutaneously injected to initiate tumor growth. Random groups (n = 8) were assigned as follows: PBS-treated, Ad-VT (9 × 10^7 PFU)-treated, Dorsomorphin (10 mg/kg)-treated, Dorsomorphin (10 mg/kg) and Ad-VT (9 × 10^7 PFU) co-treated, Raptor siRNA (15 μg)-treated, and Raptor siRNA (15 μg) and Ad-VT (9 × 10^7 PFU) co-treated groups. In the Raptor siRNA treatment group, Raptor siRNA was dissolved in 15 μL ddH2O, mixed with 7.5 μL of 10 % glucose solution and 7.5 μL of *in vivo* transfection reagent. Intratumoral injections were performed 1 day prior to Ad-VT treatment. Treatments were administered every 3 days for 6 consecutive sessions. Tumor volume was measured every 3 days over a period of 27 days, while survival was monitored daily. After 21 days of treatment, the mice were euthanized, and tumor tissues were excised, fixed in 4 % paraformaldehyde, and analyzed using TUNEL and Ki-67 staining.

### 4.14 Preparation method for dissociating xenograft tumors into single cells

After euthanizing the tumor-bearing mice, the bladder transplant tumor tissues were aseptically harvested and placed in pre-cooled PBS on ice. The tissues were rinsed with PBS, followed by removal of necrotic and connective tissues, and minced into 0.5–1 mm^3^ fragments. A 5–10 volume of trypsin digestion solution was added, and the mixture was subjected to oscillatory digestion at 37 °C for 1.5–2 h. After terminating the digestion, the cell suspension was filtered through a 50 μm cell strainer and centrifuged to collect the cells. Red blood cells were removed using red blood cell lysis buffer. The cell pellet was resuspended in complete medium (MEM supplemented with 10 % FBS and antibiotics), followed by cell counting and subsequent seeding for culture. The cells were maintained in an incubator at 37 °C with 5 % CO_2_, and the culture medium was replaced every 2–3 days [54,55].

### 4.15 Statistical analysis

All data were analyzed using GraphPadPrism version 8.0 (GraphPad Software Inc., San Diego, CA, USA) and expressed as mean ± SEM. The significance of each difference was assessed using the *t-*test or ANOVA, followed by the two-tailed *t-*test. A *p*-value of <0.05 was considered statistically significant.

## CRediT authorship contribution statement

**Dapeng Li:** Writing – original draft, Investigation, Formal analysis. **Jing Lu:** Writing – original draft, Conceptualization. **Ran Zhu:** Methodology, Data curation. **Xianyan Sun:** Resources, Methodology. **Cuiling Zhang:** Validation. **Mingzhe Sun:** Investigation. **Chengyuan Ma:** Visualization, Supervision. **Chao Shang:** Writing – review & editing, Visualization. **Xiao Li:** Visualization, Supervision, Resources.

## Consent to participate

Informed consent was obtained fromall subjects involved in the study.

## Consent to publication

Not applicable.

## Ethics approval

The animal study was reviewed and approved by the Institutional Animal Care and Use Committee (IACUC) of the Chinese Academy of Agricultural Sciences (Changchun, China).

## Declaration of competing interest

The authors declare no conflict of interest.

## Data Availability

Data will be made available on request.
