# Supplementary material for: Ad-VT oncolytic adenovirus suppresses bladder cancer via cAMP-dependent AMPK-Raptor activation and G2/M arrest
Source: Tumour Virus Res. 2026 Jan 29;21:200337. doi: 10.1016/j.tvr.2026.200337 (PMC12887817; doi:10.1016/j.tvr.2026.200337)
Supplement: Multimedia component 1 [file mmc1.docx]

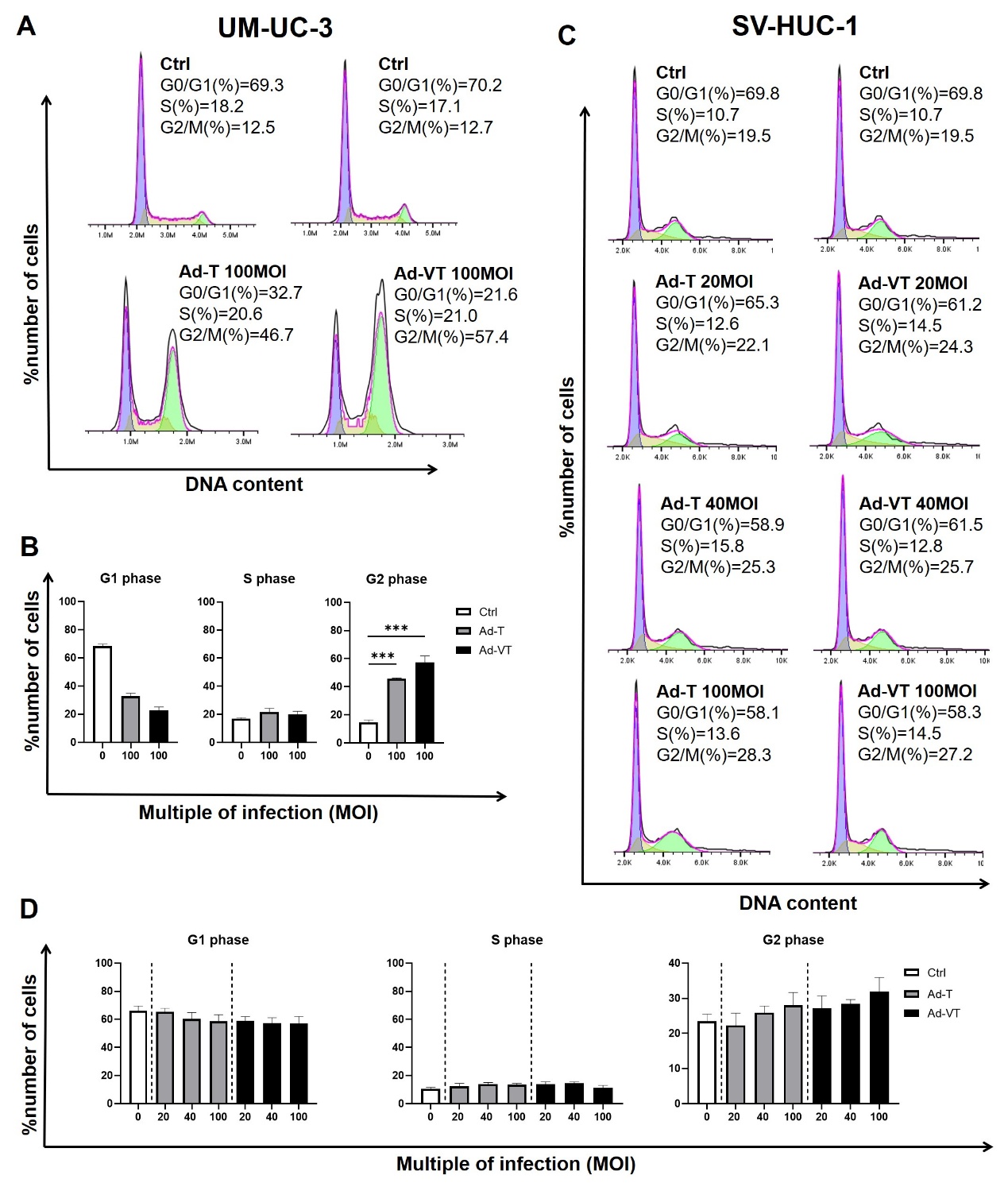


**Supplementary Figure 1 Ad-VT induces G2/M phase arrest in UM-UC-3 cells but does not affect the cell cycle progression of SV-HUC-1 cells.**

(A, B) The cell cycle distribution and analysis of UM-UC-3 cells infected with Ad-VT at 100 MOI.

(C, D) The cell cycle distribution and analysis of SV-HUC-1 cells infected with Ad-VT at different MOI.


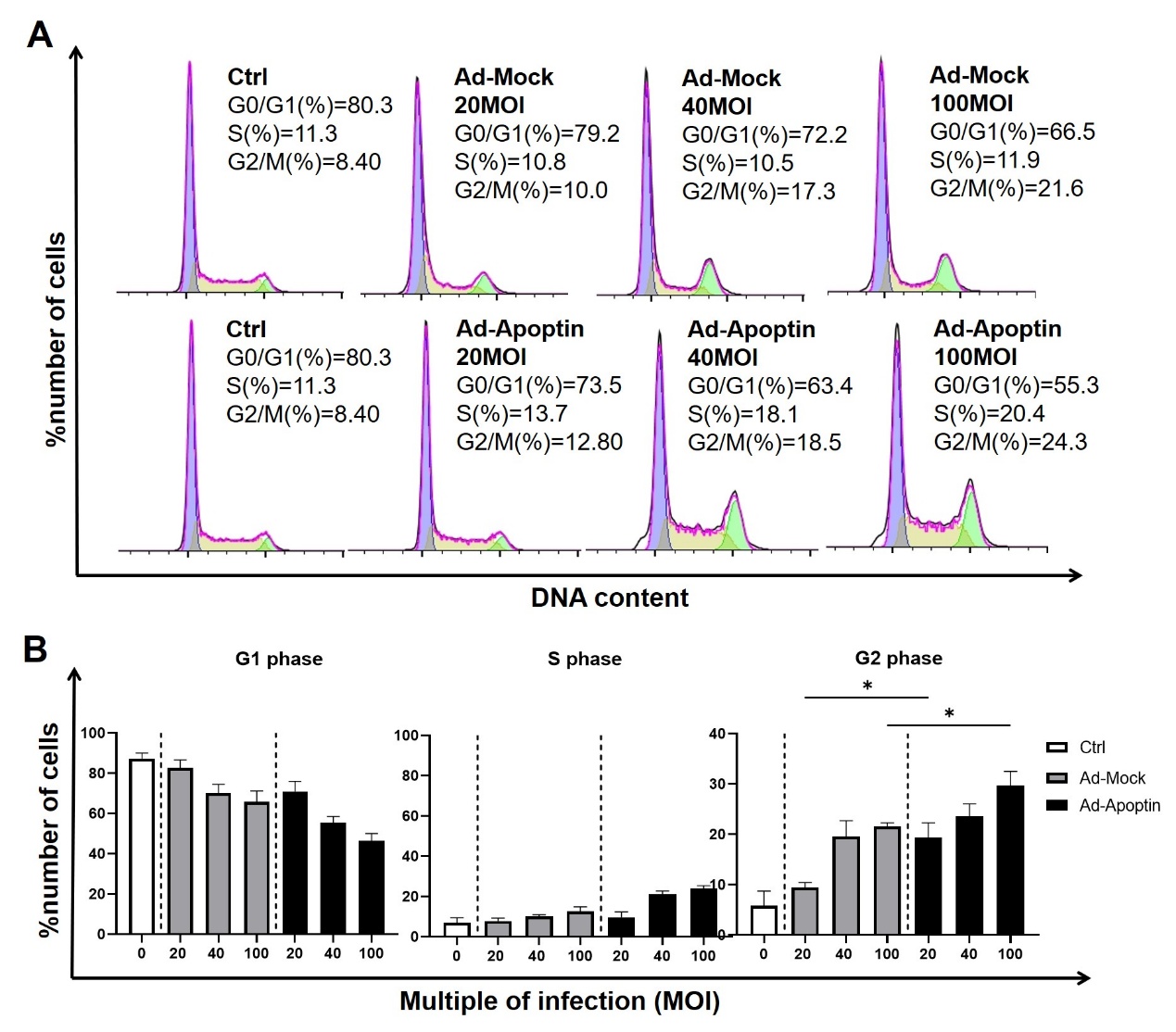


**Supplementary Figure 2 Ad-Mock and Ad-Apoptin induces G2/M phase arrest in UM-UC-3 cells.**

(A, B) The cell cycle distribution and analysis of UM-UC-3 cells infected with Ad-Mock and Ad-Apoptin a different MOI.


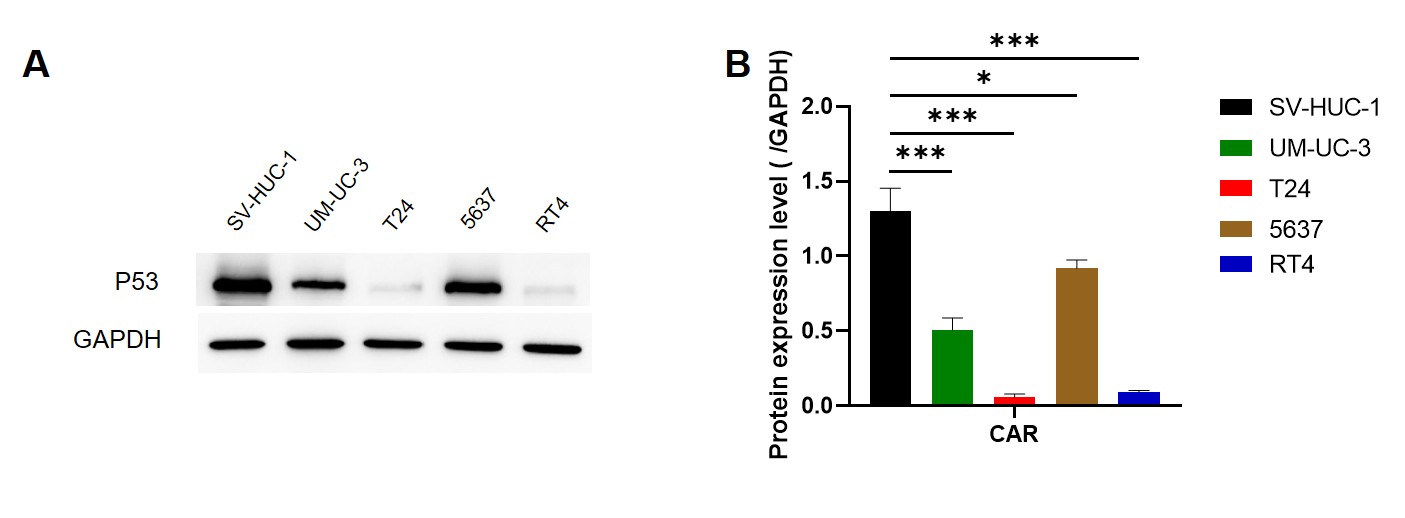


**Supplementary Figure 3 Expression levels of P53 protein in SV-HUC-1, UM-UC-3, T24, 5637, and RT4 cells**

(A, B): Expression levels of P53 protein and grayscale value analysis of protein bands in SV-HUC-1, UM-UC-3, T24, 5637, and RT4 cells.


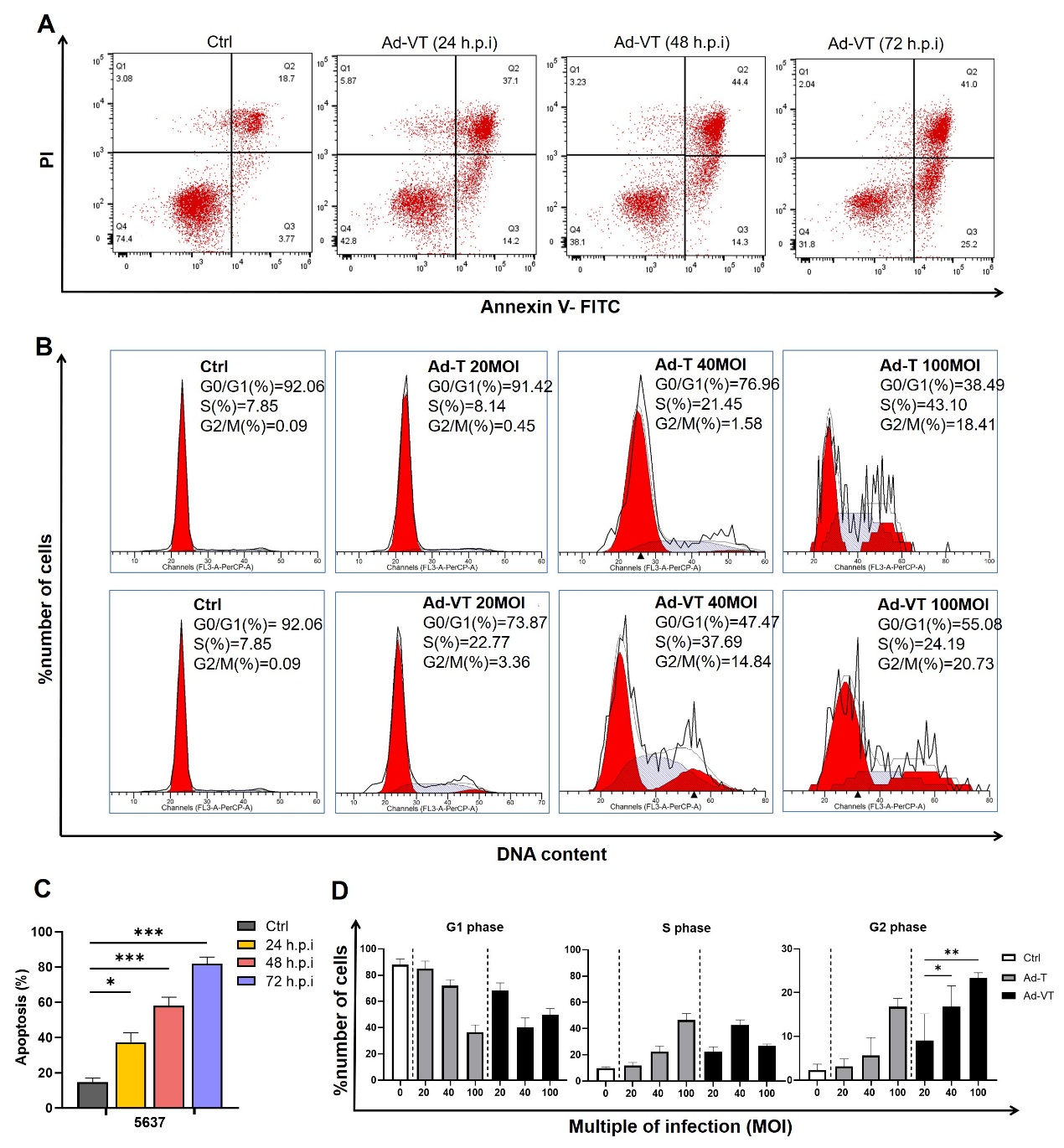


**Supplementary Figure 4 Ad-VT induces apoptosis and G2/M phase arrest in 5637 cells.**

(A, C) The percentage of apoptotic cells, as determined by flow cytometry analysis at 0, 24, 48, and 72 h after Ad-VT infection (MOI=100).

(B, D) The cell cycle distribution and analysis of 5637 cells infected with Ad-VT at different MOI.


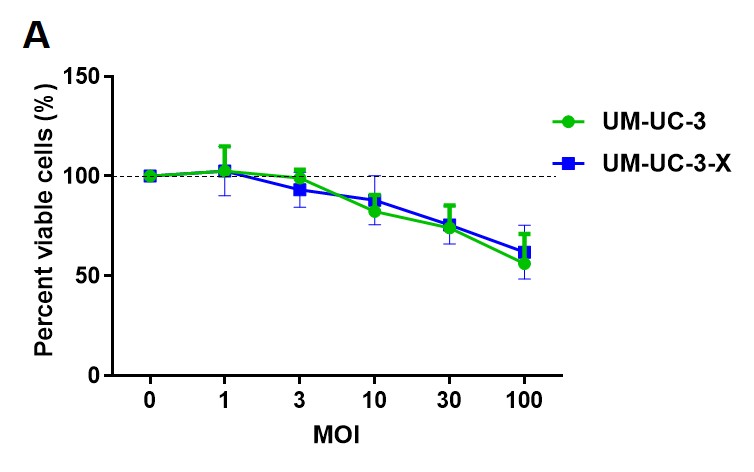


**Supplementary Figure 5 Cell Viability Assay in Xenograft Tumor Cells**

A：Cell viability was assessed using the Cell Counting Kit-8. The specified cells were infected with Ad-VT at various concentrations (MOI = 0, 1, 3, 10, 30, 100) for 48 h.
